# Preparing surgery for Generation Z - Motivation and protective factors for pursuing a surgical career among medical students and early-career physicians: a cross-sectional study

**DOI:** 10.1007/s00423-026-04296-9

**Published:** 2026-09-22

**Authors:** Runa Kinitz, Kim Klepka, Carolina Vogel

**Affiliations:** 1 Department of Trauma, Hand and Reconstructive Surgery and Orthopaedics, University Hospital Jena, Friedrich Schiller University Jena, Jena, Germany; 2 Orthopaedics University Hospital Jena, Campus Eisenberg, Eisenberg, Germany; 3 Department of Trauma and Reconstructive Surgery, University of Tuebingen, BG Trauma Center Tuebingen, Tuebingen, Germany

**Keywords:** Surgical career choice, Medical students, Gender differences in surgery, Women in Surgery, Work–life balance, Mentorship, Surgical training, Generation Z, Medical education

## Abstract

**Background:**

Surgery is facing a substantial shortage of young professionals. In particular, it has become increasingly difficult to attract medical students, especially women, to pursue a surgical career, although women currently represent the majority of medical students. The specialty is often associated with a high workload, hierarchical leadership structures, and limited compatibility between professional and private life. Against this background, the aim of this study was to identify the factors that motivate medical students, early-career physicians and board-certified surgeons to pursue a career in surgery despite these challenges.

**Methods:**

An anonymous online survey was conducted between October and December 2025. The questionnaire comprised 18 items addressing motivations as well as encouraging and discouraging factors influencing the decision to pursue a surgical career. Responses were recorded using a five-point Likert scale ranging from 1 to 5.

**Results:**

A total of 179 individuals participated in the survey (11.7% male [*n* = 21], 88.3% female [*n* = 158]; mean age 31 ± 6.3 years), including 28.5% medical students, 48.0% residents, and 23.5% board-certified surgeons. Overall, 68.7% reported having decided on a surgical career during medical school. Notably, 87.2% of respondents stated that they had been discouraged in relation to their career choice, with discouraging feedback predominantly coming from men and from individuals working in surgical specialties. Women had 6.5-fold higher adjusted odds of reporting that they had been discouraged from pursuing a surgical career compared with men (*p* < 0.001). The most frequently reported motivating factors were the operative nature of the work (97.8%), working in a supportive and appreciative team environment (60.9%), and the opportunity to make a meaningful contribution to patient care (60.9%).

**Conclusions:**

The potential future surgical workforce is predominantly female and largely belongs to Generation Z. Overall, participants emphasize the importance of high-quality and structured training, a respectful working environment, predictable career pathways, and working conditions that allow for a sustainable balance between professional and private life. Addressing these factors should be considered a strategic priority for professional societies, training supervisors, and clinical leaders to attract and sustainably retain qualified surgical trainees.

## Background

Surgical workforce shortages have become an increasing concern in healthcare systems worldwide. Several studies report a declining interest in surgical careers among medical students, particularly among women, despite the increasing representation of women in the medical workforce [1–4]. In Germany, for example, only 8.9% of female and 6.5% of male medical students report considering a surgical career at all [2]. Frequently cited reasons include demanding workloads, hierarchical workplace cultures, and limited compatibility between professional and private life, particularly during the phase of family formation [1, 3, 5].

Beyond individual career considerations, working conditions and structural challenges in surgical training have increasingly become part of broader public and professional discourse. Reports discussing deficits in postgraduate training structures, workplace culture, and hospital hierarchies have contributed to the consolidation of a negative public image of surgical careers [6–8]. This perception is further reinforced by a growing body of evidence on the psychological burden associated with surgical professions. Studies indicate that surgeons show elevated levels of psychological stress, with reported prevalence rates of depressive symptoms reaching approximately 30%, and the profession being considered a high-risk group for suicide [9, 10].

In orthopaedics and trauma surgery, this shortage is already reflected in a growing number of unfilled positions despite increasing healthcare demands in an ageing society [11]. At the same time, demographic analyses indicate a marked ageing of the surgical workforce [12]. Current national statistics show that approximately 17% of surgeons working in the inpatient sector and 37% of those working in outpatient care in Germany are aged 60 years or older. In contrast, only 21% of hospital-based surgeons and 2% of outpatient surgeons are younger than 40 years [13]. Ensuring the structured and sustainable training of new specialists is therefore not only a professional challenge but also a health policy priority for maintaining surgical care. Accordingly, the recruitment and long-term retention of young surgeons have become central topics in professional and policy discussions across digital media, print publications, and surgical conferences.

However, the decision for or against a surgical career is often made during medical school. Previous research suggests that interest in surgery may decline during medical school, particularly in the later stages of study [2]. At the same time, surveys among medical students highlight perceived barriers such as workload, hierarchical workplace cultures, and concerns about work–life balance [4, 14].

Providing medical students with early exposure to surgical disciplines and creating positive experiences during medical school therefore plays a key role in shaping career decisions.

Against this background, the aim of the present survey was to identify and systematically analyse decision-relevant factors that encourage or discourage medical students and physicians from pursuing a surgical career.

## Methods

### Study design and participants

Between October and December 2025, a cross-sectional anonymous online survey was conducted to investigate motivations as well as encouraging and discouraging factors influencing the decision to pursue a surgical career. The survey targeted medical students interested in a surgical career as well as residents and board-certified surgeons working in surgical specialties.

### Survey distribution and data collection

The survey was advertised via professional social media networks, including the channels of the German professional association Die Chirurginnen, a network supporting female surgeons. Due to the distribution via social media networks, a response rate could not be calculated.

### Questionnaire

The questionnaire was developed specifically for the present study by the research team and was reviewed and underwent an informal internal review within the author team for clarity, comprehensibility, and relevance. As it was designed to assess distinct study-specific aspects rather than a single underlying construct, formal psychometric validation and internal consistency analyses were not performed. The questionnaire consisted of 18 items covering demographic characteristics, intended or current surgical specialty, preferred work setting, and encouraging as well as discouraging factors related to the choice of a surgical career. Participants were also asked to indicate by whom discouraging experiences had occurred, including the professional role and gender of the respective individuals. In addition, protective factors that helped participants maintain their motivation to pursue surgery despite discouraging experiences were assessed. Most items were assessed using closed-ended questions and rated on a five-point Likert scale ranging from 1 to 5. Optional free-text responses were included for selected questions. The survey was administered anonymously using Google Forms. A completed questionnaire was included in the study if all questions, except for the free-text responses, had been answered.

### Statistical analysis

Data were analysed descriptively using IBM SPSS Statistics (Version 30.0, IBM Corp., Armonk, NY, USA). For descriptive analysis, Likert scale responses were aggregated into broader categories (e.g., “agree” and “rather agree”, or “disagree” and “rather disagree”) where appropriate. Categorical variables are presented as absolute and relative frequencies. Where appropriate, inferential statistical analyses were performed depending on the scale and structure of the variables.

A multivariable binary logistic regression was performed to identify factors associated with having experienced discouragement from pursuing a surgical career. The occurrence of discouragement (yes/no) was used as the dependent variable. Gender, age, professional status, and intended or current surgical specialty were included in the model. Age was entered as a continuous variable and reported per five-year increase. Surgical specialties were grouped into general and visceral surgery, orthopaedics and trauma surgery, and other surgical specialties. Adjusted odds ratios (aORs) with 95% confidence intervals (CIs) and p-values were reported. A two-sided p-value of < 0.05 was considered statistically significant. Figures were created using Microsoft Excel for Microsoft 365 (Microsoft Corp., Redmond, WA, USA).

### Ethics

This study was conducted in accordance with the principles of the Declaration of Helsinki and in line with Good Scientific Practice. Ethical approval was not required for this study, as it was an anonymous, voluntary online survey that did not involve patients, interventions, or the collection of identifiable personal data. In accordance with the General Data Protection Regulation [15], no personal data were processed. Participants were informed about the purpose of the study at the beginning of the survey, and participation was voluntary. Completion of the questionnaire was considered as provision of informed consent.

## Results

### Demographic characteristics

A total of 179 individuals participated in the survey, including 11.7% men (*n* = 21) and 88.3% women (*n* = 158). The mean age of participants was 31 ± 6.3 years. Among respondents, 28.5% were medical students (mean age 25.2 ± 3.0 years; 2.8% in the preclinical phase, 12.3% in the clinical phase, and 13.4% during the final year of medical school), 48.0% were residents (mean age 30.6 ± 3.7 years), and 23.5% were board-certified surgeons (mean age 39.1 ± 5.1 years). Participants reported their intended specialty (medical students) or current specialty (residents and board-certified surgeons). Overall, 34.1% were associated with orthopaedic and trauma surgery and 30.7% with general and visceral surgery. The remaining respondents were distributed across other surgical disciplines (Table 1).

**Table 1:** Distribution across surgical specialties

Surgical specialty	*n*	%
Orthopaedics and trauma surgery	61	34.1
General and visceral surgery	55	30.7
Plastic surgery	13	7.3
Cardiac and thoracic surgery	13	7.3
Vascular surgery	7	3.9
Paediatric surgery	7	3.9
Gynecology	5	2.8
Urology	4	2.2
Neurosurgery	2	1.1
Otorhinolaryngology	2	1.1
Oral and maxillofacial surgery	2	1.1
No specification	8	5.6
**Total**	**179**	**100.0**

### Preferred work setting

**Fig. 1:**
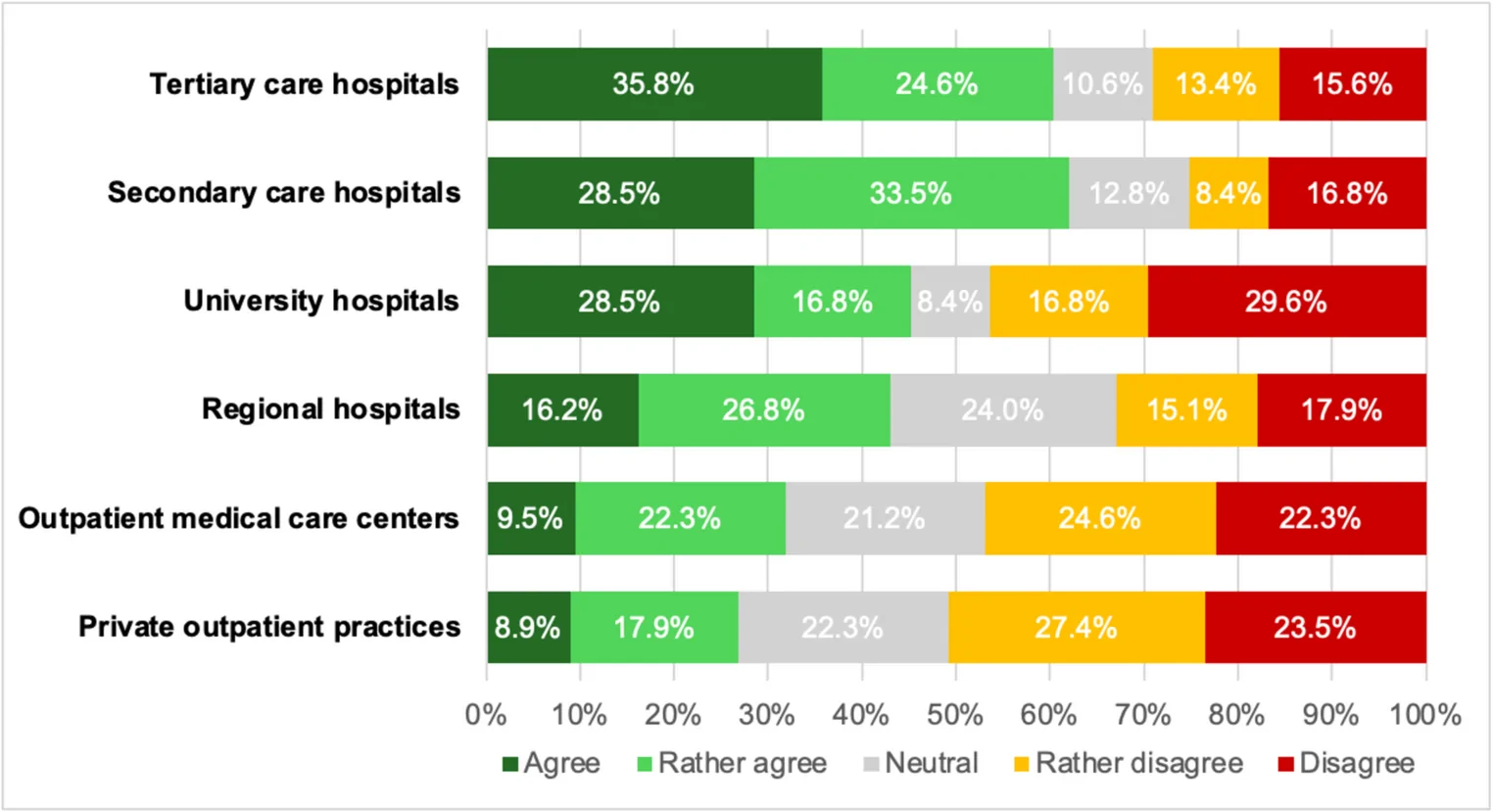
Preferred workplace settings for a future or current surgical career (*n* = 179)

Overall, participants expressed a clear preference for working in the hospital sector (Fig. 1). The highest levels of agreement (“agree” or “rather agree”) were reported for tertiary care hospitals (60.4%) and secondary care hospitals (62.0%). University hospitals were preferred by 45.3%, and regional hospitals were preferred by 43.0% of respondents. An outpatient medical care center was preferred by 31.8% of participants, whereas 46.9% rejected this option. Similarly, 26.8% of respondents were open to working in a private outpatient practice, while more than half (50.9%) rejected this option (Fig. 1).

### Timing of the decision to pursue a surgical career

Overall, 68.7% of respondents reported that they had decided to pursue a surgical specialty during medical school (10.6% during the preclinical phase, 31.8% during the clinical phase, and 26.3% during the final year). For 27,9% of participants, the decision to pursue a surgical career had already been made before entering medical school. A further 2.2% reported deciding in favour of surgery during a different residency training program, while 1.2% did not provide an answer.

### Encouraging factors

**Fig. 2:**
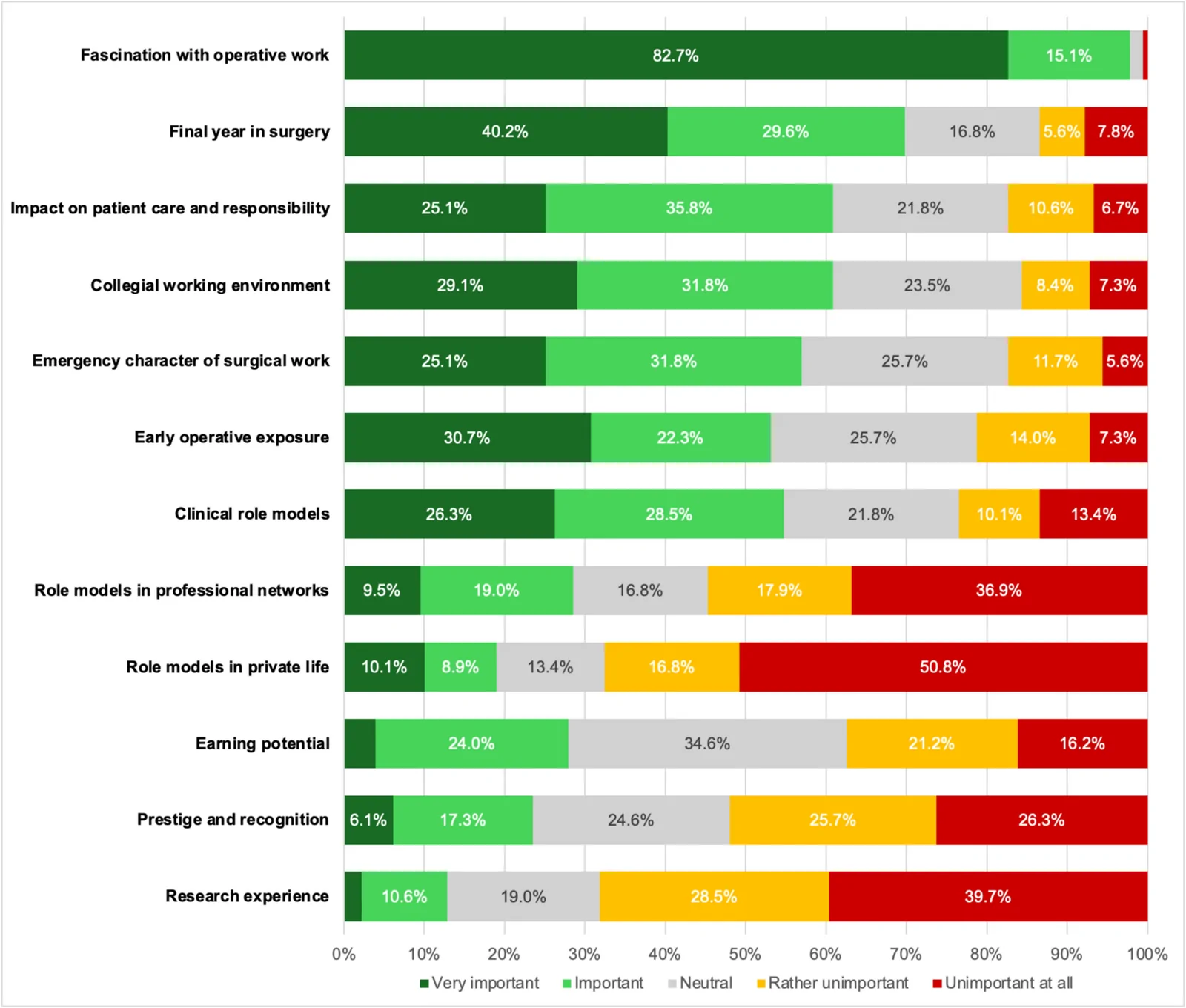
Encouraging factors influencing the decision to pursue a surgical career (*n* = 179). Values < 5% are not labeled for clarity

Intrinsic motivations emerged as the most important encouraging factors for pursuing a surgical career. Fascination with operative work was rated as “very important” or “important” by the vast majority of respondents (82.7% and 15.1%, respectively) (Fig. 2). Similarly, the opportunity to have a meaningful impact on patient care and to assume responsibility was considered important by many participants (25.1% “very important”, 35.8% “important”). The emergency character of surgical work showed comparable levels of agreement (25.1% “very important”, 31.8% “important”). A positive team environment and collegial working culture were also frequently reported as motivating factors (29.1% “very important”, 31.8% “important”). Experiences during medical training played an additional role. The final year of medical school in surgery was rated as important by 40.2% (“very important”) and 29.6% (“important”) of respondents, while early operative exposure during medical school was rated as important by 30.7% (“very important”) and 22.3% (“important”). One participant summarized this experience in a free-text response:

- *“During my final year, I encountered several wonderful surgeons. One of the department heads combined outstanding surgical skills with empathy toward both colleagues and patients, which made me decide that this was exactly the kind of surgeon I wanted to become” (translated from German).*

Role models also influenced career decisions. Clinical role models were rated as important by 26.3% (“very important”) and 28.5% (“important”) of respondents, whereas role models in professional networks and private life were less frequently perceived as relevant. In contrast, extrinsic incentives were considered less important. Research experience was rated as “rather unimportant” or “not important at all” by most respondents (28.5% and 39.7%, respectively). Similarly, prestige and professional recognition (25.7% “rather unimportant”, 26.3% “not important at all”) and earning potential (21.2% “rather unimportant”, 16.2% “not important at all”) played a comparatively minor role.

### Discouraging factors

**Fig. 3:**
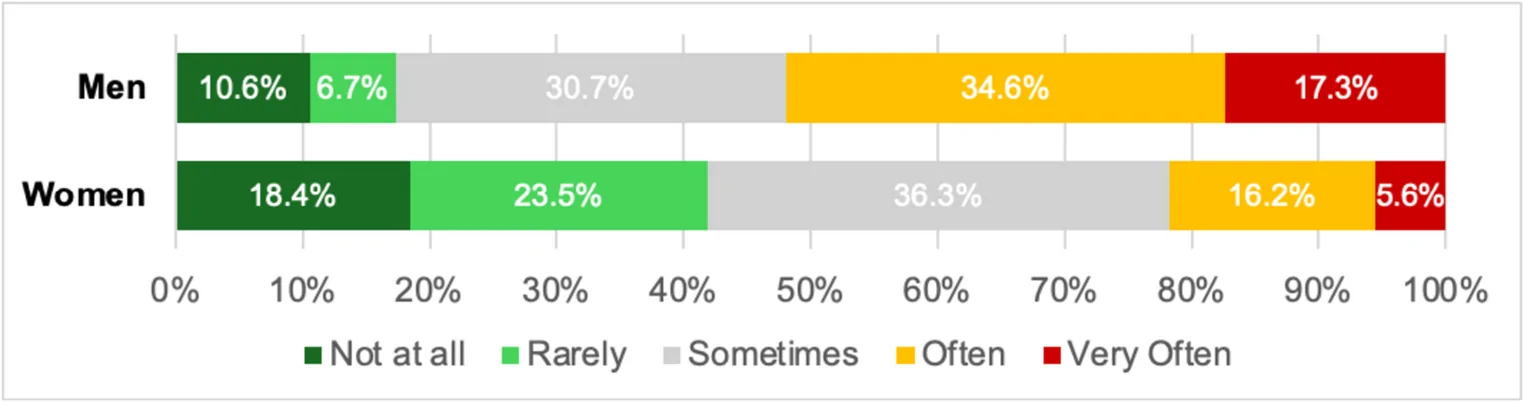
Frequency of discouraging feedback according to the gender of the person expressing discouragement (*n* = 179)

Overall, 87.2% of respondents reported that they had at some point been discouraged from pursuing a surgical career. Men were significantly more likely than women to give discouraging feedback (*p* < 0.001) (Fig. 3). While discouragement from women was more often described as occurring “rarely” or “sometimes,” discouragement from men was more frequently reported as occurring “often” or “very often.”

**Fig. 4:**
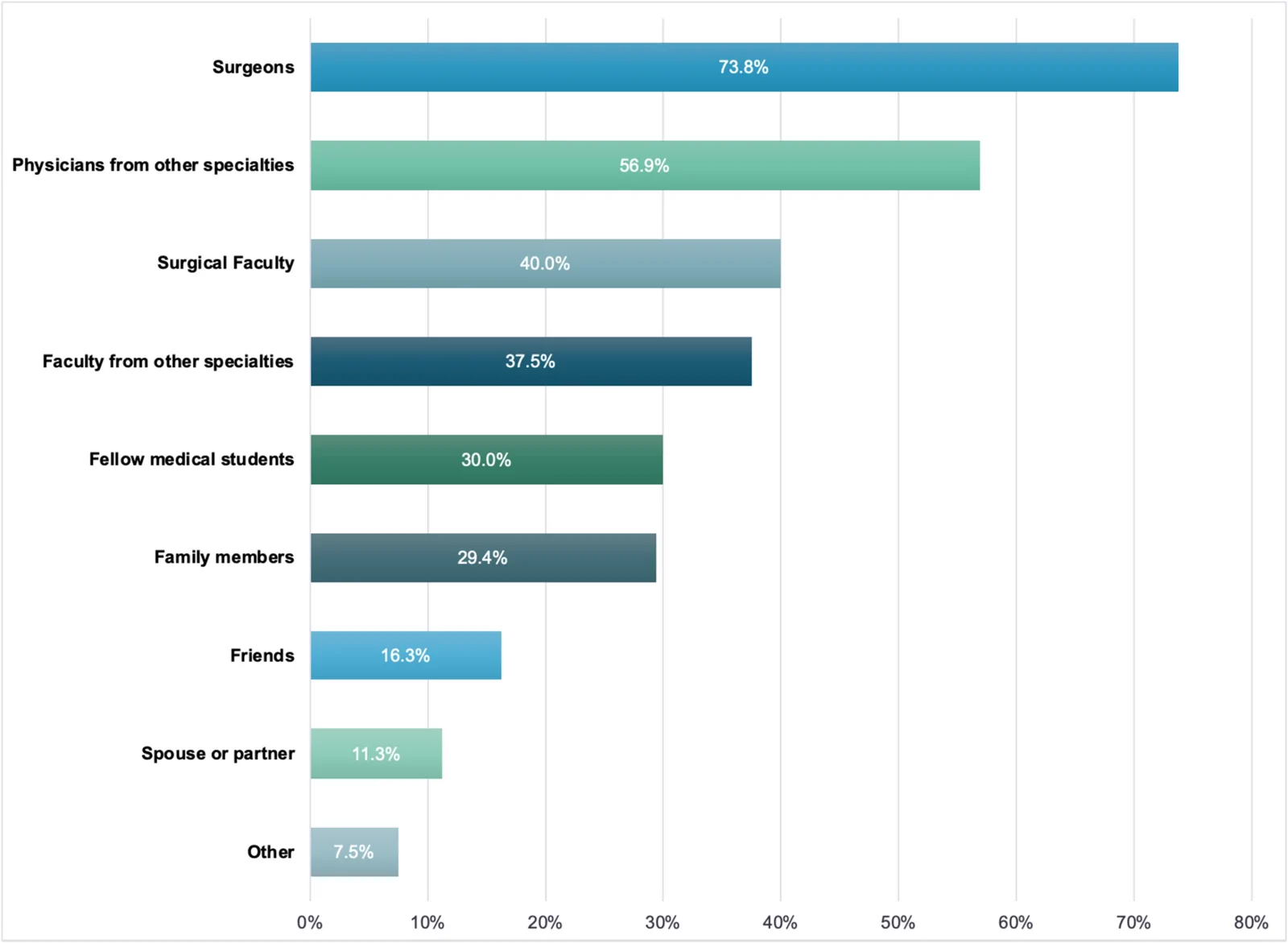
Sources of discouraging feedback when considering a surgical career. Respondents indicated from whom discouraging comments had been received. Multiple responses were possible (*n* = 160 respondents/484 responses)

Among respondents who reported discouragement, 73.8% stated that this feedback had come from surgeons themselves (Fig. 4). In addition, 56.9% reported having been discouraged by physicians from other specialties, and 40.0% indicated that discouraging comments had been made by surgical lecturers. One participant summarized these experiences in a free-text response as follows:

- *“Whenever I mentioned my intention to pursue a surgical specialty, many physicians—often from other disciplines—seemed to feel obliged to talk me out of it” (translated from German).*

In the multivariable logistic regression analysis, gender was independently associated with having experienced discouragement from pursuing a surgical career. Women had significantly higher odds of reporting discouragement than men (aOR 6.45, 95% CI 2.18–19.13; *p* < 0.001). No independent associations were observed for age, professional status, or intended or current surgical specialty (Table 2).

**Table 2:** Multivariable binary logistic regression analysis of factors associated with having experienced discouragement from pursuing a surgical career

Predictor	aOR (95% CI)	*p* value
Women vs. men	6.45 (2.18–19.13)	*< 0.001*
Age, per five-year increase	0.92 (0.52–1.63)	*0.779*
Residents vs. medical students	1.60 (0.43–5.92)	*0.479*
Board-certified surgeons vs. medical students	0.66 (0.09–5.05)	*0.689*
General and visceral surgery vs. other surgical specialties	0.96 (0.30–3.08)	*0.939*
Orthopaedic and trauma surgery vs. other surgical specialties	0.85 (0.27–2.63)	*0.773*

*aOR* adjusted odds ratio, *CI* confidence interval

Outcome: experienced discouragement (yes vs. no); *n* = 179

### Structural reasons for discouragement

**Fig. 5:**
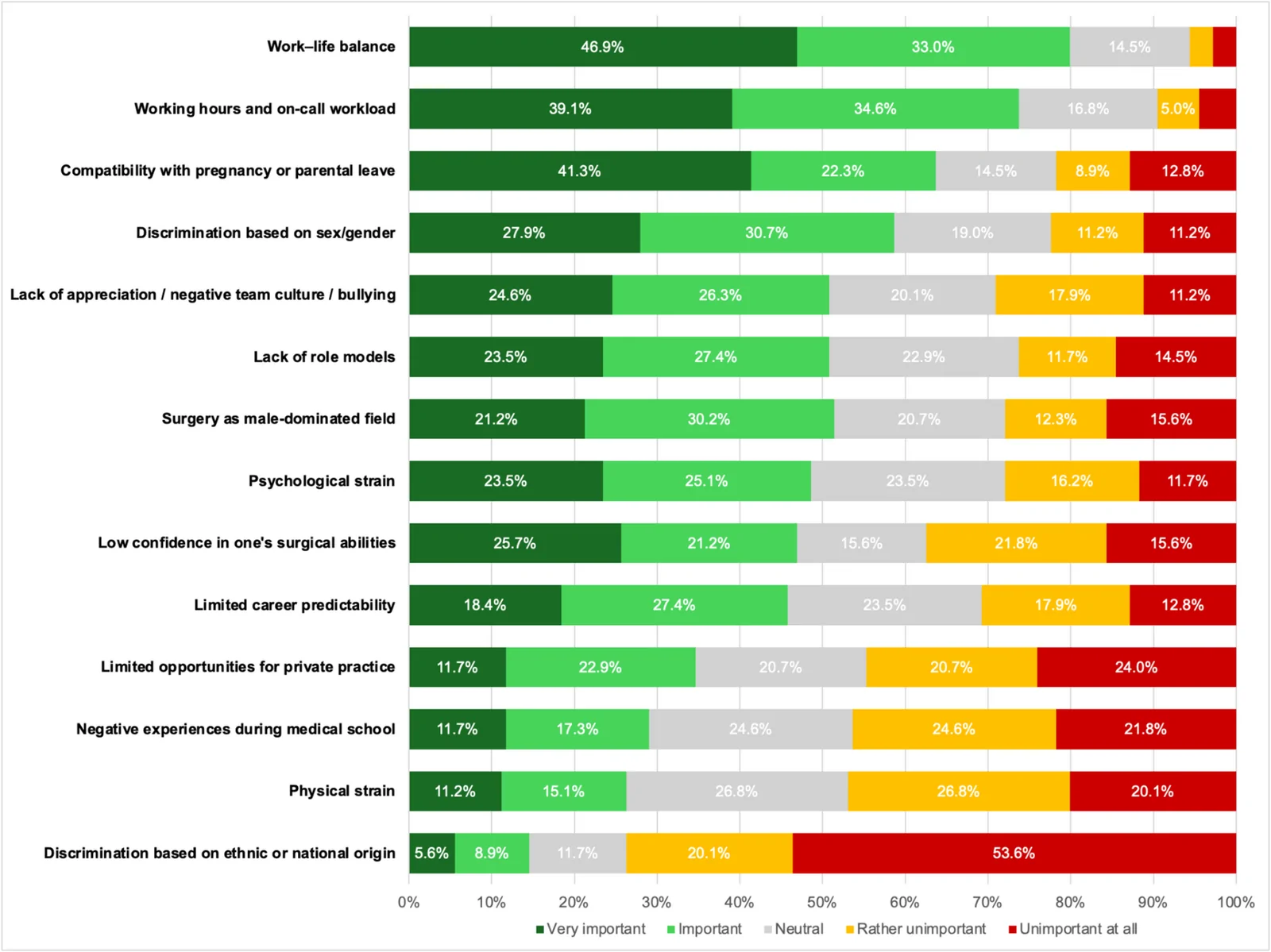
Structural factors contributing to discouragement when considering a surgical career (*n* = 179). Values < 5% are not labeled for clarity

The survey further examined structural factors that may contribute to discouragement when considering a surgical career and their perceived importance (Fig. 5). The most frequently reported discouraging factors were the perceived lack of work–life balance (very important 46.9%, important 33.0%) and demanding working hours and on-call workload (very important 39.1%, important 34.6%). Similarly, the perceived incompatibility of surgical training with pregnancy or parental leave was rated as highly relevant (very important 41.3%, important 22.3%). Further discouraging factors included a lack of appreciation or negative team culture (very important 24.6%, important 26.3%), the perception of surgery as a male-dominated field (very important 21.2%, important 30.2%), and a lack of role models (very important 23.5%, important 27.4%). In contrast, physical strain associated with operative work (not important at all 20.1%, rather unimportant 26.8%), negative experiences during medical school (not important at all 21.8%, rather unimportant 24.6%), and limited opportunities for private practice (not important at all 24.0%, rather unimportant 20.7%) were less frequently perceived as discouraging factors.

### Protective factors

**Fig. 6:**
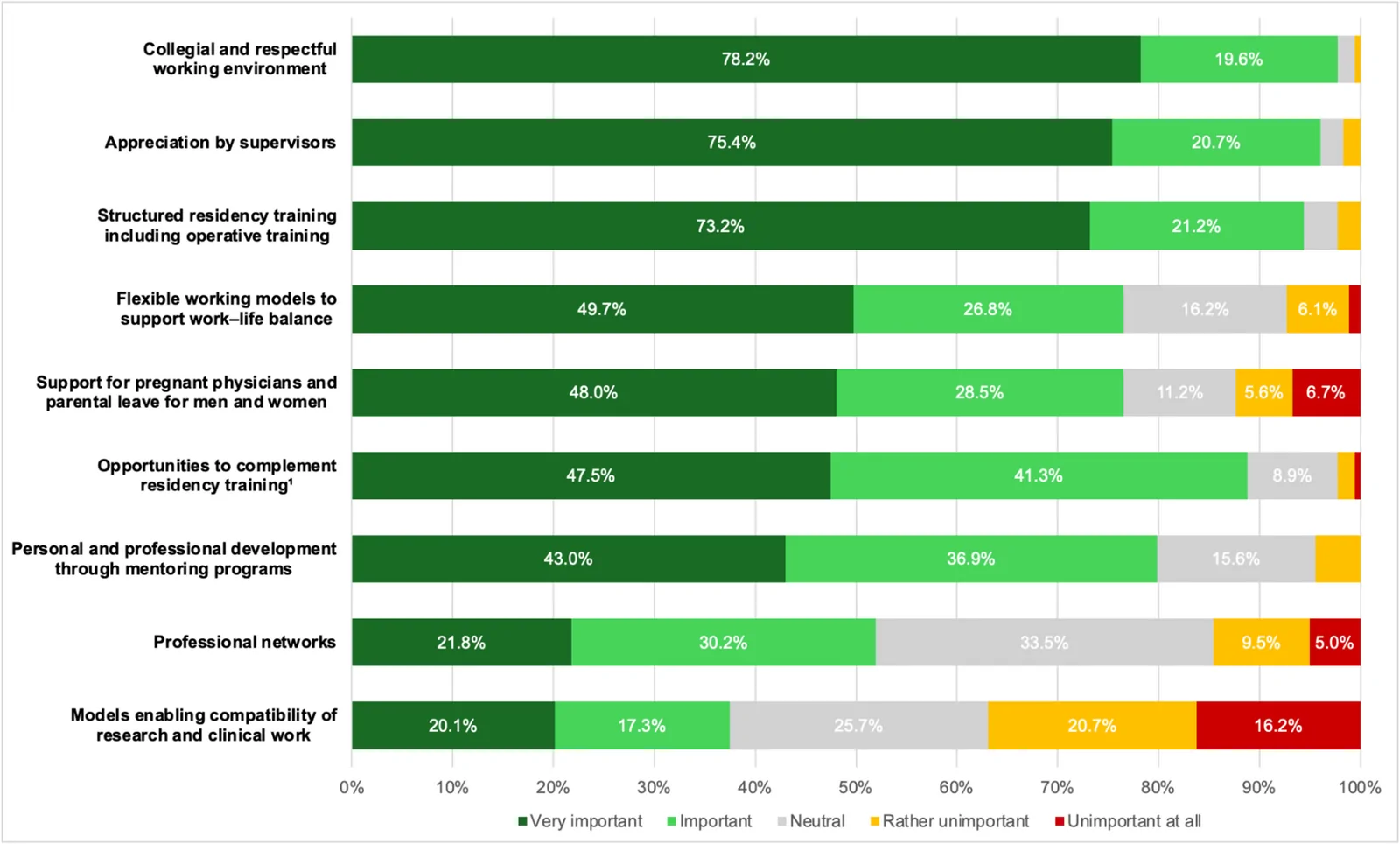
Protective factors supporting motivation to pursue and maintain a surgical career (*n* = 179). ¹ e.g., courses, workshops, access to simulation training, conference participation. Values < 5% are not labeled for clarity

Participants were also asked which factors help maintain their motivation for a surgical career (Fig. 6). The most important protective factors were a collegial and respectful working environment (very important 78.2%, important 19.6%) and appreciation by supervisors (very important 75.4%, important 20.7%). A structured residency training including operative training was also rated highly (very important 73.2%, important 21.2%). Flexible working models supporting work–life balance (very important 49.7%, important 26.8%) and institutional support for pregnancy and parental leave for both women and men (very important 48.0%, important 28.5%) were also frequently identified as important protective factors. Opportunities to complement residency training (very important 47.5%, important 41.3%) and mentoring programs supporting personal and professional development (very important 43.0%, important 36.9%) were rated positively as well. In contrast, professional networks (very important 21.8%, important 30.2%) and models enabling compatibility of research and clinical work (very important 20.1%, important 17.3%) were considered less important by respondents.

## Discussion

The present survey provides insight into factors that encourage and discourage surgical career motivation among surgically interested medical students and surgeons in Germany, with particular relevance to the emerging Generation Z workforce. A clear majority of respondents (68.7%) reported that they had decided in favor of a surgical career during medical school, particularly during the clinical phase and the final year. This is notable because previous national data show that interest in surgery tends to decline during medical school. While around 35% of students can imagine a career in surgery at the start of their studies, this proportion decreases to approximately 23% in the clinical years and to 19.3% in the final year [2]. A survey of around 1,000 medical students likewise showed that the high workload perceived by more than 85% of respondents represents a major barrier to pursuing a surgical career. In addition, students perceive surgery as strongly hierarchical and characterized by a harsh tone, which further reduces its attractiveness [4, 14]. This image stands in clear contrast to what medical students expect from their future profession, with “team” being one of the most important factors [2]. In our survey, a collegial and respectful working environment as well as appreciation by supervisors emerged as the most highly weighted protective factors for choosing and maintaining a surgical career.

### Training quality and structural conditions

A second major finding of this study was the high importance attributed to structured surgical training. Overall, 94.4% of respondents considered structured surgical training to be important or very important. At the same time, operative teaching and the completion of required rotations are frequently perceived as insufficient, not least due to increasing workload compression under unchanged staffing conditions [16]. As a result, the same clinical workload must be managed within shorter time frames, while opportunities for operative training are increasingly displaced by administrative demands [17].

In this context, digital transformation may offer part of the solution. The implementation of intelligent hospital information systems and innovative digital infrastructures may improve job satisfaction, optimize clinical workflows, and reduce bureaucratic burden, particularly for residents [18–20]. At the same time, such developments may also create better conditions for reconciling professional and private life [20].

### Work–life balance, gender, and career sustainability

The perceived incompatibility of surgery with private life and family responsibilities remains a central issue. In the present survey, a lack of work–life balance and demanding working hours were among the most important discouraging factors. However, because the study population was predominantly female, these findings cannot be interpreted as evidence of gender-specific differences. Nevertheless, the idea that surgery cannot be reconciled with private life, particularly in the context of family formation and care work, is widespread. Yet compatibility between work and private life is the single most important professional expectation among medical students, with 92.5% rating it as very important [2]. Given persistent societal role expectations, in which women continue to assume the majority of caregiving responsibilities [21], it is unsurprising that the negative image of surgery may disproportionately discourage female medical students from pursuing a surgical career [4, 22–25].

Accordingly, male students are more likely than female students to consider a surgical career (19% vs. 11%) [14]. Similar patterns have also been reported in orthopaedics and trauma surgery, where around 21% of medical students in the early semesters—regardless of gender—can imagine a career in the field, but interest among women declines over the course of medical school while remaining largely stable among men [3]. Given that women now represent around two-thirds of medical students, surgery appears to be failing to fully utilize a major recruitment pool due to persistent structural barriers. Consequently, women remain underrepresented in surgical specialties, both clinically and academically [26–28].

Against this background, the career dip or even dropout of women from surgical career paths appears almost structurally embedded in rigid working-time and career models, as the demanding phase of surgical residency frequently coincides with the typical period of family formation [4, 24, 26, 29]. The implementation of flexible working models, family-friendly departmental structures, childcare support, and transparent career pathways that remain compatible with parenthood should therefore be seen as a key strategy for attracting and retaining surgical staff, regardless of gender.

### Generation Z and the appeal of surgery

The medical student subgroup, with a mean age of 25.2 years, predominantly fell within the age range commonly attributed to Generation Z (born between 1993 and 2012) [30].

In discussions about the shortage of young surgeons, this generation is sometimes portrayed as less resilient or less willing to commit to demanding careers. However, there is no robust evidence to support this assumption. On the contrary, Generation Z has been described as highly engaged, strongly value-driven, and willing to take responsibility—qualities that are in fact highly compatible with surgery [30, 31]. The present findings support this interpretation. Respondents placed high value on social responsibility toward patients, and fascination with operative work and manual skills emerged as the strongest motivating factor for entering surgery (97.8%). These findings suggest that the challenge is not a lack of interest in surgery itself, but rather a mismatch between the values of the next generation and the working conditions they perceive within the specialty [30, 31].

### The importance of early exposure and teaching

Passion for surgery can be fostered through curricular and extracurricular teaching formats, which may create important opportunities for targeted recruitment. This has increasingly been recognized by universities, professional societies, and surgical associations, which have developed practical and theoretical programs specifically aimed at students. Such initiatives may help communicate the fascination of surgery in an authentic and constructive way [32]. Another possible recruitment strategy is the integration of medical students as assistants in surgical departments, which may positively influence career choice [33]. At the same time, the final year remains a particularly important phase for later specialty choice [33]. This was also reflected in our survey, in which the surgical final-year rotation was rated as an encouraging factor by the majority of participants. Since all students in Germany rotate through surgery during the final year, this phase represents a particularly important window for shaping career decisions. It should therefore be understood not only as a final stage of undergraduate training, but also as a strategic opportunity for sustainable recruitment into surgical specialties [34, 35].

### Discouragement from within surgery

One of the most striking findings of this study is that discouragement often came from within surgery itself. Overall, 87.2% of respondents reported that they had already been discouraged in relation to their surgical career choice. Women in the present sample had higher adjusted odds of reporting discouragement than men. However, this finding should be interpreted cautiously because only 21 men participated and the confidence interval was wide. It therefore cannot be generalized as a population-level gender difference. Discouraging feedback was reported from both women and men, although men were named more frequently. Most notably, discouraging feedback most often came from surgeons themselves. Given the widespread dissatisfaction with structural working conditions in surgery, this is understandable [10]. From the perspective of workforce development, however, it is clearly counterproductive. Potentially motivated candidates may be deterred from entering the field, even though they might later contribute to improving the very structures they are being warned about. For this reason, improving the public and professional image of surgery is essential. That image is inseparably linked to the attitudes and behavior of surgeons in both teaching and everyday clinical practice. Structural reform is necessary, but it will not automatically change external perceptions of the field. Surgery also needs a more active culture of communication, visible role modeling, and credible advocacy if persistent stereotypes are to be overcome.

### Mentoring and cultural change

In this context, structured mentoring may be particularly valuable. In both our survey and the literature, mentoring is described as a relevant factor supporting the decision to pursue a surgical career [22, 25]. This appears especially important for women, for whom mentors, sponsors, and allies may be critical in navigating structural barriers and career transitions [24, 36]. In surgery, mentoring can serve multiple functions: long-term personal and professional career guidance, targeted operative support, access to networks, and support for academic development [37, 38]. Mentoring has also been associated with positive effects on individual competence, psychosocial resources, and organizational outcomes, including reduced stress and burnout [37]. Mentoring should therefore be understood not merely as an individual support measure, but as a strategic instrument for sustaining the future surgical workforce. In a period of structural transformation, mentoring may help maintain enthusiasm for surgery while fostering a more inclusive, professional, and future-oriented surgical culture.

### Limitations

This study has several limitations. First, participants were recruited primarily through social media and the authors’ professional networks, including their involvement in Die Chirurginnen e.V., a professional association for women in surgery. This recruitment strategy resulted in a highly selected, self-recruited sample comprising 88.3% women and a substantial proportion of respondents with an existing interest in surgery or surgical specialties. The proportion of women even exceeded that among medical students in Germany, approximately two-thirds of whom are women [39]. Consequently, male students, students without a particular interest in surgery, and individuals not reached through these networks may be underrepresented. The findings therefore cannot be considered representative of Generation Z medical students in Germany and should not be interpreted as population-level prevalence estimates. Selection bias may also have affected the observed attitudes toward surgical careers and working conditions, although the direction and magnitude of this effect cannot be determined.

The high proportion of women in this survey contrasts sharply with the current proportion of women in surgery in Germany, which is only 23.3% [13]. Consequently, the findings likely reflect a disproportionately female perspective on the factors for and against choosing surgery. At the same time, this may also be considered a strength of the survey, as it captures in detail the perspectives of Generation Z women, whose recruitment and long-term retention are essential to securing future surgical care.

Second, the survey did not undergo formal external or psychometric validation. Therefore, measurement error and differences in the interpretation of individual items cannot be excluded, and the findings should be considered exploratory. However, the survey included only medical students interested in surgery as well as surgeons already working in surgical disciplines. No comparison group of students or physicians without surgical interest was included. This introduces substantial selection bias and limits the generalizability of the findings. Future studies should therefore include participants from other specialties in order to better understand which specific factors discourage individuals from surgery and which structural or cultural barriers are perceived. Nevertheless, the explicit focus of the present study was on identifying factors that motivate people to choose surgery. These positively framed influences provide valuable starting points for targeted recruitment strategies and may offer constructive impulses for the further development of the field.

Third, the participants in the survey are either working or undergoing training within the german healthcare system. As working and training conditions, as well as regulations concerning, for example, training structures and maternity protection, differ considerably across healthcare systems internationally, the generalizability of the findings to other healthcare systems is limited.

## Conclusions

The present survey offers a predominantly female Generation Z perspective on motivating and discouraging factors related to surgical career choice. Against the background of a medical student population in which women represent the majority, the findings identify several modifiable factors that may improve both recruitment into surgery and long-term retention within the specialty through the modernization of infrastructure, workplace culture, and leadership. In summary, future surgeons seek excellent training, a respectful and appreciative team environment, reliable career pathways, and working conditions that allow professional and private life to coexist sustainably. Addressing these issues should therefore be understood as a strategic responsibility of professional societies, training supervisors, and clinical leaders. A consistent structural and cultural modernization of surgery may help strengthen the specialty’s attractiveness and secure its long-term future in the competition for qualified young professionals.

## Data Availability

No datasets were generated or analysed during the current study.
